# Mandibular Involvement in Recurrent Multifocal Osteomyelitis Associated with SAPHO Syndrome

**Published:** 2018-01

**Authors:** Igor-Moreira Hazboun, Thiago-Pires Brito, Vanessa-Gonçalves Silva, Carlos-Eduardo-Monteiro Zappelini, Leopoldo-Nizam Pfeilsticker

**Affiliations:** 1 *Department of Otolaryngology Head and Neck, State University of Campinas, Faculty of Medical Sciences, Campinas, Sao Paulo, Brazil.*; 2 *Otorhinolaryngologist, Coordinator of Ambulatory Surgery and Maxillofacial Traumatology, State University of Campinas, Faculty of Medical Sciences, Sao Paulo, Brazil. *

**Keywords:** Mandibular involvement, Osteomyelitis, SAPHO syndrome

## Abstract

**Introduction:**

SAPHO syndrome is defined as the association of a group of rare sterile osteoarticular disorders and inflammatory skin diseases whose etiology, although not yet determined, probably involves genetic, immunological and infectious mechanisms. The recurrent multifocal osteomyelitis, an inflammatory disease, can be associated with this syndrome even as a single event.

**Case Report:**

A case of a young female patient, with a definite diagnosis of SAPHO and an inflammatory mandibular atypical disease for which therapeutic options with immunosuppressants were being used, is reported. The adverse evolution of the clinical conditions led to the hypothesis that the patient suffered from associated mandibular odontogenic bacterial osteomyelitis. The extraction of all teeth was recommended. After our evaluation, we recommended a conservative treatment, and after 2 months of treatment with an endovenous antibiotic, the patient showed improvement of clinical and laboratory results.

**Conclusion:**

Early diagnosis is necessary to avoid successive and unnecessary tooth loss in the treatment of chronic osteomyelitis mandibular.

## Introduction

The SAPHO syndrome (synovitis acronym, acne, pustulosis, hyperostosis and osteitis) was described in the 80s by Chamot and defines the association of a rare group of sterile inflammatory bone-joint disorders with skin diseases (1). With an estimated prevalence of 1: 10,000, the bone-joint manifestations represent a diagnostic and therapeutic challenge since they may precede skin changes in years (2,3). 

The recurrent multifocal osteomyelitis, which involves the mandible, is an inflammatory disease characterized by recurrent episodes of intense regional pain often accompanied by trismus, paresthesia and progressive deformity of the mandible and can be an isolated manifestation of SAPHO syndrome (4,5). 

The mandibular osteomyelitis in SAPHO syndrome is an aseptic phenomenon and can usually be treated conservatively with the use of immunosuppressive agents. The occurrence of bone kidnappings and abscesses is not part of the syndrome and it raises suspicion for bacterial mandibular osteomyelitis, which may be treated with antibiotic therapy (6). 

The aim of this report is to warn physicians about the relationship between chronic mandibular osteomyelitis and SAPHO syndrome. In those cases, the use of antibiotics as a sole therapy may fail, leading to extreme attitudes and a compromised prognosis. The extensive knowledge of this syndrome and its various manifestations is necessary for proper therapeutic management. 

## Case Report

A female patient, of 27 years, was diagnosed with SAPHO syndrome and nonspecific combined immune deficiency when she was six. She presented with chronic recurrent multifocal osteomyelitis as the main manifestation of the syndrome, including involvement of the mandible. Since the age of six, multiple biopsies on the patient, especially in the ankle joint, confirmed the presence of aseptic osteomyelitis. Prescribed with a recurrent use of pamidronate, the patient had sequels like osteoporosis and growth failure caused by steroids being continuously maintained at varying doses. 

Four years ago, she began to complain of mandibular pain, which was intermittent and did not cease with painkillers and anti-inflammatory steroids. At this time, she also underwent bone scans showing intense mandibular osteoclastic activity. 

Since then, the patient presented with several clinical and laboratory signs of inflammatory activity of the underlying disease. Several unsuccessful therapeutic approaches have been attempted, such as thalidomide and azathioprine, which is associated with corticosteroids. Due to the persistence of the active disease, the use of pamidronate was suspended two years ago when monoclonal antibody infliximab was introduced. 

Nine months ago, the mandibular pain got significantly worse and restriction of the mouth opening and chewing was noted. At the time, the patient was using steroids in addition to infliximab. The adverse evolution of the clinical condition, paired with the worsening of inflammatory evidences and the finding of febrile peaks led to the hypothesis that the patient was suffering from bacterial mandibular osteomyelitis associated with dental foci. 

**Fig 1:**
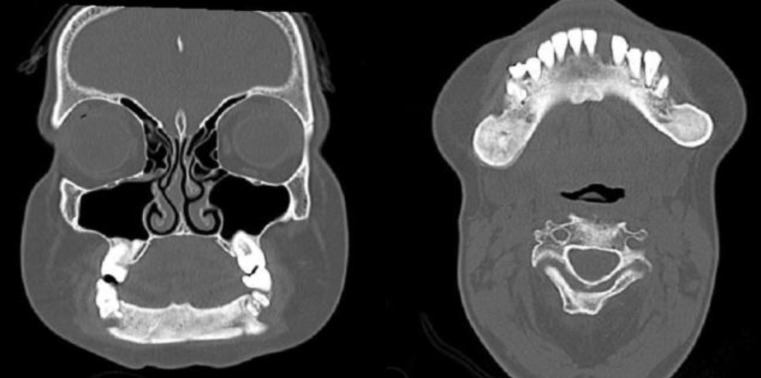
Face CT scan with bone windowing in coronal and axial sections showing homogeneous diffuse bone sclerosis of the mandible, with fat obliteration of bone marrow without cortical thickening

**Fig 2:**
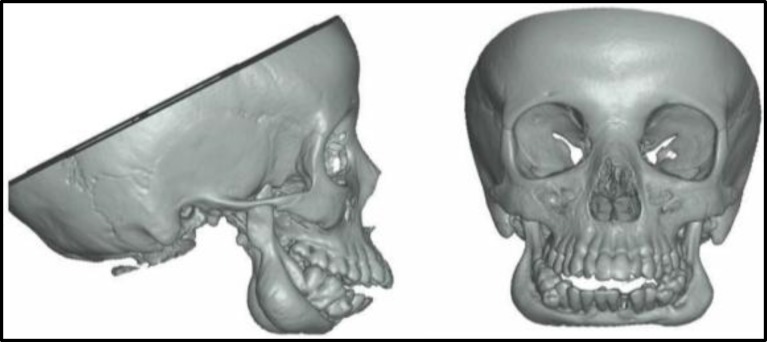
Three-dimensional facial reconstruction showing diffuse bone sclerosis of body and branches of the mandible associated with bilateral hypoplasia condyle and retrognathia

The patient’s CT scan of the face showed relatively homogeneous bone sclerosis, diffuse body and branches of the mandible, fat obliteration of the bone marrow without cortical thickness, bilateral condylar hypoplasia, and retrognathia (Figs.1,2). 

**Fig3:**
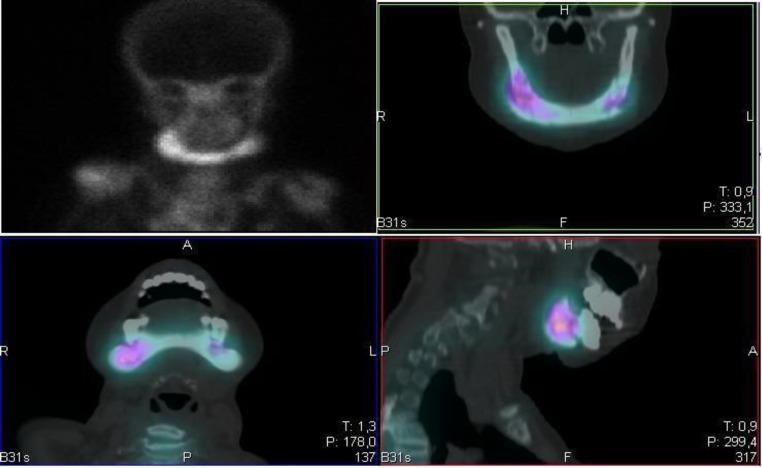
Bone scintigraphy with technetium-99m showing increased blood flow to the mandible, with diffuse heterogeneous uptake of the radiopharma- ceutical in marked degree to the fullest extent of the mandible, compatible with osteomyelitis in activity

The bone scintigraphy SPECT/CT with technetium99m showed increased blood flow to the mandible. It also showed diffuse heterogeneous uptake of the radiopharma- ceutical in marked degree to the fullest extent of the mandible, compatible with osteomyelitis in activity (Fig.3). Observed changes in the shoulders, humeral head, and hip joints were already present in a previous study and remained unchanged. 

A dental evaluation was requested, revealing generalized periodontitis, pulp vitality and grade 3 mobility of all mandibular teeth. A complete mandibular extraction and debridement of the periodontal region was suggested. Fearing the increased risk of fracture by the presence of mandibular bone thinning, it was requested that we assess the patient and jointly perform the proposed procedure. However, after detailed analysis and multidisciplinary discussion, we proposed a conservation treatment because of the chronic nature of SAPHO syndrome and the frequent association of diffuse osteomyelitis sclerosing mandible. After two months of antibiotic intravenous therapy (amoxicillin+ clavulanate), the clinical condition of the patient and the laboratory tests improved. After antibiotic treatment, the patient continued to use infliximab for 12 months associated with oral corticosteroids. The patient reported no pain. It was also observed that she had grade 1 mobility in some dental elements, no periodontitis and showed satisfactory mouth opening and chewing. Under these conditions, we tried to terminate corticosteroid treatment. However, the recurrence of pain led to the resumption of the drug. 

## Discussion

SAPHO syndrome was proposed as a term for a group of conditions with similar Musculoskeletal manifestations. In particular, these conditions include the hyperostosis of the anterior chest wall, the synovitis and aseptic multifocal osteomyelitis observed in association with skin conditions such as palmoplantar pustulosis, severe acne, and hidradenitis suppurativa (7). This condition is rare and is characterized by intermittent episodes of painful arthritis which can be disabling. Patients with SAPHO usually have musculoskeletal complaints. However, the diagnosis can be difficult in cases where there is only bone involvement without skin lesions (8). 

The etiology of the syndrome is still unclear, but probably involves genetic, immunological and infectious mechanisms. The potential pathogenic role of infectious agents in genetically predisposed individuals was only suggested once. Microorganisms such as Propionibacterium acnes (bacteria associated with acne) were detected in bone biopsies (9). 

SAPHO syndrome can present a favorable intermittent course in most cases. However it can also present as a chronic course, which is associated with progressive and with debilitating pain and requires aggressive treatment approaches. 

Chronic osteomyelitis diffuse sclerosing of the mandible could be a unique osteoarticular manifestation of SAPHO syndrome. The involvement of the mandible is found in almost 10% of syndrome cases, and all patients should have full body scans in the search for other inflammatory foci. Late diagnosis of SAPHO in cases of mandibular osteomyelitis can lead to ineffective or inadequate treatments and serious sequelae such as temporomandibular ankylosis, which often requires aggressive surgical procedures which could normally be prevented (10,11). The classification of mandibular osteomyelitis as bacterial osteomyelitis or as osteomyelitis associated with SAPHO syndrome is recommended (6). The diagnostic criteria for bacterial osteomyelitis are suppuration and osteolytic change. Mandibular osteomyelitis in SAPHO syndrome is characterized by non-suppurative involvement and radiographic changes in periosteal lamellar type reaction, external bone resorption and enlargement of the bones. 

Currently, because there is no cure, the treatment of this syndrome is still empirical and focused on symptom control. Various treatment regimens with many drugs (including anti-inflammatory drugs, systemic corticosteroids, sulfasalazine, methotrexate, cyclosporin, leflunomide, calcitonin) are often unable to control the disease and expose patients to their side effects. There was a significant improvement in disease control with the use of derivatives of bisphosphonates and tumor necrosis factor α anti-(anti-TNF α) (12). SAPHO syndrome cases successfully handled by pamidronate and zoledronic acid, bisphosphonates and infliximab (antiTNF-α) have been reported in the literature (13,14). In a severe case of SAPHO, although the treatment with adalimumab (anti-TNF-α) has determined after 48 weeks to complete clinical remission of the cutaneous and osteo-articular manifestations, a follow up with imaging tests revealed progression of osteoarticular involvement with maintained osteitis (15). According to Garcovich et al, TNF-α antagonists have been used as a third-line therapy but there are few studies with long-term follow-up (15). A case of SAPHO with diffuse sclerosing osteomyelitis of the mandible was successfully treated with prednisone and bisphosphonates by Hatano et al (16). Other cases and small series have reported good results after intravenous treatment with bisphosphonates. The bone specific alkaline phosphatase (a marker of bone formation) and cross-linked pyridinoline telopeptide of type I collagen carboxy (marker for bone resorption) showed a marked decrease with the use of pamidronate (17). Oxygen therapy in a hyperbaric chamber is also described as a therapeutic option, but it is still controversial (18). Despite the occurrence of mandibular osteonecrosis as a side effect in the prolonged use of oral bisphosphonates for non-oncologic indications, it is well defined and should be considered (19). It is also known that prolonged use of corticosteroids is the leading cause of non-traumatic osteonecrosis (20). However, our scintigraphy investigation showed no significant change in hip condition, which is the most commonly affected spot in these circumstances. We must consider that after more than 15 months of pamidronate suspension, the clinical condition was still evolving unfavorably, and after stabilization, any removal of steroids promoted pain return. Thus, the current clinical condition maintained in our patient for more than 12 months indicates that, for now, the synergic action of infliximab and corticosteroids is favorable for the patient. 

In rare cases, surgery would be more effective than a conservative treatment. In general, surgical intervention may be suggested when there is limited mandibular function such as lesions affecting the temporomandibular joint, limiting the mouth opening, and causing varying degrees of ankylosis. Other indications would be cosmetic disfigurement, progressive pain and the failure of conservative therapy (8). Wide resection of the mandibular bone and immediate reconstruction with microvascular flaps is one of the surgical procedures used. Surgery is usually a last resort once the results are temporary with recurring symptoms. 

We emphasize that chronic osteomyelitis diffuse sclerosing of the mandible must be distinguished from microbial osteomyelitis with suppuration, a phenomenon that can coexist and is often confused with the first one. Bacterial osteomyelitis is easily cured by antibiotic treatment. On the other hand, the isolated use of those drugs is generally ineffective when sclerosing chronic osteomyelitis and SAPHO syndrome are present. 

SAPHO syndrome’s natural evolution is associated with exacerbations and remissions, leading to debilitating conditions with persistent pain. Few patients will have a self-limited course of the disease. For this reason, although the patient could be stable after a year, it’s important to keep tracking the patient for an indefinite period because of the strong possibility of recurrence of the mandibular frame. 

## Conclusions

The functional outcome in the treatment of mandibular chronic osteomyelitis associated with SAPHO syndrome depends on early diagnosis and individualized therapeutic management. Therapies with multiple immunomodulatory agents and antibiotics, in the presence of associated infections, can minimize sequelae, avoiding unnecessary invasive procedures such as surgery and tooth avulsion. 
